# Evaluation of microbial consortia and chemical changes in spontaneous maize bran fermentation

**DOI:** 10.1186/s13568-017-0506-y

**Published:** 2017-11-16

**Authors:** Marilù Decimo, Mattia Quattrini, Giovanni Ricci, Maria Grazia Fortina, Milena Brasca, Tiziana Silvetti, Federica Manini, Daniela Erba, Franca Criscuoli, Maria Cristina Casiraghi

**Affiliations:** 1 Institute of Sciences of Food Production, National Research Council of Italy, Milan, Italy; 2 Department of Food, Environmental and Nutritional Sciences, University of Milan, Milan, Italy

**Keywords:** Maize bran valorization, Spontaneous fermentation, Microbial consortia, Lactic acid bacteria, Yeasts, Bioactive compounds

## Abstract

Sustainable exploitation of agro-industrial by-products has attracted great interest in cereal bran valorization. In this research, a polyphasic approach has been carried out to characterize maize bran at microbiological and chemical level during a sourdough like fermentation process, in order to enhance its technological and nutritional properties. Autochthonous microbiota was isolated at different refreshment steps and subjected to identification and molecular characterization. Fermentation was characterized by a rapid increase in lactic acid bacteria and yeasts, with a co-dominance, at the initial stage, of *Weissella* spp., *Pediococcus* spp. and *Wickerhamomyces anomalus.* At the end of the fermentation, a natural selection was produced, with the prevalence of *Lactobacillus plantarum*, *Lactobacillus brevis* and *Kazachstania unispora.* This is the first time that a specific association between LAB and yeasts is reported, during the maize bran fermentation process. Enzymatic activities related to this microbial consortium promoted a “destructuration” of the fiber fraction, an increase in soluble dietary fiber and a reduction of phytic acid content. Our data also evidenced a noticeable increment in ferulic acid. The results obtained indicate that fermentation processes represent an efficient biotechnological approach to increase nutritional and functional potential of maize bran. Moreover, the characterization of microbiota involved in natural fermentation process will allow the selection of specific biotypes, with appropriate metabolic and enzymatic activities, to conduct “tailored” fermentation processes and improve brans or whole-meal flours from both nutritional and technological points of view.

## Introduction

Cereal bran valorization has, for many reasons, a noticeable interest. The dietary recommendations call for an increased intake of functional foods: by-products generated by cereal agro-industrial sector are rich in dietary fiber, minerals and bioactive compounds and could represent beneficial ingredients for human nutrition. The main reasons behind the low utilization of native brans as ingredient for cereal-based products are due to their poor safety (heavy metal and mycotoxins), technological (increased dough stickiness, reduced volume) and sensory qualities (dark color, taste, chewy/hard texture) (Rose et al. 2010; Heinio et al. 2016). The spontaneous sourdough-like fermentation process, characterized by a consortium of yeasts and lactic acid bacteria (LAB), has shown to be an interesting pre-treatment in order to ameliorate sensory and textural quality, as well as to improve the microbial safety of bran (Dalié et al. 2010; Manini et al. 2014; Messia et al. 2016). Recently, several studies have shown the ability of many LAB to inhibit mold growth and mycotoxin biosynthesis (Dalié et al. 2010). These data highlight the potential offered by LAB as natural agents for decontamination of food frequently contaminated by toxigenic fungal strains, particularly cereals. Moreover, through fermentation, an increased bioavailability of bioactive compounds and a decreased level of some anti-nutritional compounds can be obtained (Katina et al. 2012; Manini et al. 2014).

To date, the majority of published literature in this field regards fermented wheat bran. Little is known about the effects of this “bio-approach” on properties of others cereal, as maize. This cereal and its by-products could represent attractive alternative raw materials for fiber enrichment of gluten-free products. According to A.I.R.E.S (Associazione Italiana Essiccatori Raccoglitori Stoccatori di Cereali e Semi oleosi, http://www.micotossine.it/), about 82% of maize production is used for animal feed and only 18% is devoted to human consumption. At the milling stage, dry maize mechanical processing creates whole or fractionated products, accounting about for up to 25%, generally used as animal feed or biofuel. In this context, the valorization of maize by-products as valuable food ingredients to be exploited in the production of new food formulation could represent an opportunity.

Ferulic acid is diffused in cereals and maize bran appears as one of the richer sources, containing about 26–33 g/kg (Zhao and Moghadasian 2008). Moreover, maize bran contains hemicellulose, cellulose and a small amount of lignin, thus representing a source of dietary fiber. The hemicellulosic fraction, generally denominated arabinoxylans (AX), has a particular heterogeneous nature, with extensive cross-linkages between ferulic acid and arabinose/xylose heteropolisaccharides. This particular feature promoted a complex and rigid structure of maize cell wall (Rose et al. 2010). The potential of native microorganisms and their enzymatic activities of increasing the fraction of soluble dietary fibers and the content of free ferulic acid deserves to be tested.

Recently the EFSA Panel published a positive Opinion about the request of an Health Claim related to the positive effects on the post-prandial glycemic and insulinemic responses elicited by water-extractable arabinoxylans (WEAX) (EFSA 2011). This Opinion was founded on results from different studies reporting that this fraction can delay the rate of carbohydrates digestion/absorption, thus positively affecting glucose metabolism (Ai and Jay-lin 2016; EFSA 2011). Moreover, it is quite recognized in literature that arabino-xylanoligosaccharides (AXOS) and xylan-oligosaccharides (XOS) obtained by the hydrolysis of arabinoxylans present in brans can exert prebiotic properties (Broekaert et al. 2011).

Little data are available in literature on microbial population during maize fermentation. They mainly refer to maize-based spontaneously fermented doughs produced in West African countries (Oguntoyinbo et al. 2011; Okeke et al. 2015; Assohoun-Djeni et al. 2016). Very lacking information exists on microbial population during maize bran fermentation and on their contribution in promoting nutritional and functional properties of this cereal by-product. Thus, this study was performed to characterize maize bran, at microbiological and chemical level during a sourdough like fermentation process.

## Materials and methods

### Fermentation process and chemical analyses

Two commercial native maize brans (C1 and C2—average particle size 500–600 µm—Molino Perteghella, Solarolo di Goito, MN, Italy; Molino Spoletini, Arcevia, AN, Italy) were subjected to spontaneous fermentations (without microbial starters), according to Manini et al. (2014). Fermentation experiments were carried out in duplicate, at 30 °C, through continuous propagation until a stable microbiota was established (12 days). Unfermented samples and fermented bran samples, collected at different refreshment steps were stored at − 20 °C for further analysis.

Proximate composition, ferulic acid, total (TOTAX) and water-soluble (WEAX) arabinoxylans, were assessed, in duplicate, on native and fermented maize brans as previously reported (Manini et al. 2014).

### Evaluation of microbial population

Using selective media and conditions as previously reported (Manini et al. 2014), we quantified and isolated lactic acid bacteria (LAB), non-lactic acid bacteria (NLAB), yeasts and molds.

For each fermented bran, between 5 and 20 colonies representing all morphologies were picked from the respective plates at the refreshment steps 1, 7 and 12, purified by successive streaking, and stored in glycerol at − 80 °C for further experimentations. A total of 135 presumptive LAB, 93 yeast isolates, 12 NLAB and three molds were selected and subjected to identification and molecular characterization.

### Molecular identification of the isolates

Total bacterial DNA was extracted from 100 µL of an overnight culture, using the Microlysis kit (Labogen, Rho, Italy) following the manufacturer’s instructions. For LAB identification, a first clustering step was reached by a PCR amplification of the *16S*–*23S* rDNA spacer region (RSA). Molecular identification of LAB isolates with different RSA patterns, and NLAB was carried out by partial *16S* rDNA gene sequencing, species-specific amplification and/or restriction analysis of the *16S*–*23S* rDNA spacer region. For the isolated yeasts and molds, total DNA was extracted in a PRECELLYS^®^24-DUAL lyser/homogeniser (Bertin-technologies, Saint Quentin en Yvelines, France) (3 cycles of 30 s, 30-s break) with a mixture of glass beads (Ø < 106 µm). Molecular identification was carried out by restriction digestion of the Internal Transcribed Spacer (ITS) and/or partial 26S rDNA gene sequencing. The DNA sequences for the primers used, their corresponding specificities and the thermal cycle parameters employed are reported in Table 1. Amplification was carried out in a Mastercycler (Eppendorf, Hamburg, Germany). PCR was performed in a 25 μL reaction mixture containing 50–100 ng DNA template, 2.5 µL 10× reaction buffer (Thermo Fisher Scientific, Vilnius, Lithuania), 200 µmol L^−1^ of each dNTP, 2.5 mmol L^−1^ MgCl_2_, 0.5-mmol L^−1^ of each primer, and 0.5-U *Taq* polymerase (Thermo Fisher Scientific). The amplification products were separated on a 1.5–3.0% agarose gel stained with ethidium bromide and photographed. Amplicons were purified using NucleoSpin^®^ Extract II (Macherey–Nagel, Düren, Germany) and sequenced at Eurofins Genomics (Ebersberg, Germany). Sequence alignment was carried out with ClustalW software. The BLAST algorithm was used to determine the most relate sequence relatives in the National Centre for Biotechnology Information nucleotide sequence database (http://www.ncbi.nlm.nih.gov/BLAST). The *16S* rRNA gene restriction digestion was carried out employing *Alu*I, *Cfo*I, and *Dra*I (Thermo Fisher Scientific), according to the supplier’s instructions. The products of the PCR-amplified Internal Transcribed Spacers ITS region were digested with the restriction endonucleases *Hin*fI and *Taq*I. Restriction digests were subsequently analyzed by agarose electrophoresis, as above reported.

**Table 1:** PCR primers and conditions used for isolates identification

Primers	Target	Sequence (5′–3′)	Thermal conditions	Amplicon (bp)	References
RSA_F RSA_R	16S–23S rDNA spacer region (RSA)	GAAGTCGTAACAAGG CAAGGCATCCACCGT	94 °C × 45 s 54 °C × 1 min × 35 cycles	Variable	Lane (1991)
16S_F 16S_R	*16S* rDNA gene	AGAGTTTGATCCTGGCTCAG CTACGGCTACCTTGTTACGA	94 °C × 45 s 55 °C × 45 s × 35 cycles	1540	Lane (1991)
p8FLP p806R	Partial *16S* rDNA gene	AGTTTGATCCTGGCTCAG GGACTACCAGGGTATCTAAT	94 °C × 1 min 56 °C × 1 min × 30 cycles	800	McCabe et al. (1995)
*L. plant*_F *L. plant*_R	*L. plantarum*	CCGTTTATGCGGAACACC TCGGGATTACCAAACATCAC	94 °C × 2 min 56 °C × 1 min × 35 cycles	318	Torriani et al. (2001)
Pedio23S_F Pedio23S_R	*Pediococcus* spp.	GAACTCGTGTACGTTGAAAAGTGCTGA GCGTCCCTCCATTGTTCAAACAAG	94 °C × 45 s 64 °C × 1 min × 35 cycles	701	Pfannebecker and Fröhlich (2008)
P23S_R PPE23S_F	*P. pentosaceus*	CTGTCTCGCAGTCAAGCTC CCAGGTTGAAGGTGCAGTAAAAT	94 °C × 1 min 67 °C × 1 min × 35 cycles	1640	Pfannebecker and Fröhlich (2008)
P23S-R ArgentF	*P. argentinicus*	CTGTCTCGCAGTCAAGCTC GATATTCCTGTACTAGTTAGAT	94 °C × 1 min 60 °C × 1 min × 35 cycles	948	This study
Lbrev 1391F	*L. brevis*	TAATGATGACCTTGCGGTC TGTACACACCGCCCGTC	94 °C × 45 s 48 °C × 45 s × 35 cycles	330	Coton et al. (2008)
Weiss_F Weiss_R	*Weissella* spp.	CGTGGGAAACCTACCTCTTA CCCTCAAACATCTAGCAC	94 °C × 45 s 54 °C × 1 min × 35 cycles	725	Jang et al. (2002)
1 RL LaccreR	*L. lactis*	TTTGAGAGTTTGATCCTGG GGGATCATCTTTGAGTGAT	94 °C × 2 min 54 °C × 1 min × 35 cycles	238	Pu et al. (2002)
LeuclacF LeuclacR	*L. lactis*	AGGCGGCTTACTGGACAAC CTTAGACGGCTCCTTCCAT	94 °C × 2 min 58 °C × 1 min × 35 cycles	742	Lee et al. (2000)
ITS1 ITS4	ITS1-5.8S-ITS2 Internal Transcribed Spacer (ITS)	TCCGTAGGTGAACCTGCGG TCCTCCGCTTATTGATATGC	94 °C × 45 s 60 °C × 1 min × 35 cycles	Variable	Jespersen et al. (2005)
NL1 NL4	*26S* rDNA gene	GCATATCAATAAGCGGAGGAAAAG GGTCCGTGTTTCAAGACGG	94 °C × 2 min 52 °C × 1 min × 35 cycles	778	Kurtzman and Robnett (1998)

### Molecular fingerprinting of lactic acid bacteria

LAB strains were typed by random amplification of polymorphic DNA-PCR (RAPD) typing with primers M13 (5′-GAGGGTGGCGGTTCT-3′) and LP1 (5′ACGCGCCCT-3′). An annealing temperature of 38 and 48 °C for M13, and LP1 respectively, and an amplification protocol of 35 cycles were used. The PCR products were analyzed by electrophoresis and photographed as reported earlier. Grouping of the RAPD-PCR profiles was obtained with the BioNumeric 5.0 software package (Applied Maths, Kortrijk, Belgium), following the unweighted pair-group method with arithmetic averages cluster analysis. The value for the reproducibility of the assay, evaluated by the analysis of repeated DNA extracts of representative strains was > 93%.

### Carbohydrate utilization

The utilization of different carbon sources was carried out using a basal medium (containing Peptone 1,5% wt/v, Yeast extract 0.6% wt/v, chlorophenol red 0.004% wt/v, Tween 80 1 ml L^−1^, pH 6.4) and the desired filter-sterilized carbohydrate at a final concentration of 0.5% (wt/v).

### pH measurement

Measurement of pH was determined on 10 g of fermented bran suspended in 100 mL of distilled water by a pH meter (PHM 250, Radiometer, Copenhagen, Denmark).

### Statistical analysis

All data are reported as mean ± standard deviation. One-way ANOVA was performed on chemical data; if significant *(p* < 0.05) effects were found, pairwise comparisons between samples were checked with Tukey’s test (Statgraphics XV version 15.1.02, StatPoint Inc., Warrenton, VA, USA).

### Nucleotide sequence accession numbers

The *16S* rDNA sequences determined during the present study have been deposited in the GenBank database under the accession numbers MF348245–MF348248 and MF399041–MF399049. The *26S* rDNA sequences determined during the present study have been deposited in the GenBank database under the accession numbers MF348249–MF348254.

## Results

### Microbiological analyses

In this study, two commercial native maize brans were used to prepare spontaneous laboratory sourdoughs. The values of microbial counts as well as the pH during fermentation are shown in Table 2. Fermentation was characterized by a rapid increase in LAB number after the first day of bran fermentation, reaching levels of 10^8^–10^9^ CFU/g, for both bran samples; these levels remained constant during all refreshment steps. The trend for the yeasts paralleled those of the LAB population throughout fermentation. From an initial concentration of 10^2^–10^3^ CFU/g an increasing and gradual growth was observed, reaching levels of 10^7^–10^8^ CFU/g, after 3–5 days of fermentation. The count was stable until the end of fermentation. The data obtained indicated that, even if the LAB count stayed higher than that of yeasts, LAB and yeasts had similar growth throughout the fermentation. Contrarily to these fermenting microorganisms, the molds population, with initial load of 10^4^ CFU/g, decreased rapidly after 2–3 days of fermentation.

**Table 2:** Viable cell counts and pH values during spontaneous fermentation of maize bran C1 and C2

Days	Maize bran C1					Maize bran C2				
	NLAB	Yeasts	Moulds	LAB	pH	NLAB	Yeasts	Moulds	LAB	pH
0	4.5 ± 0.2	2.5 ± 0.7	3.7 ± 0.1	3.6 ± 0.6	5.1 ± 0.1	3.3 ± 0.1	2.0 ± 0.5	4.0 ± 0.1	3.1 ± 0.3	5.1 ± 0.1
1	8.8 ± 0.3	4.0 ± 0.4	2.9 ± 1.3	8.8 ± 0.1	4.0 ± 0.1	7.8 ± 0.2	5.5 ± 0.4	4.7 ± 0.1	8.1 ± 0.1	5.2 ± 0.1
2	8.4 ± 1.3	5.0 ± 0.4	3.8 ± 0.3	9.0 ± 0.2	3.7 ± 0.1	7.8 ± 0.3	7.8 ± 0.3	2.0 ± 1.3	9.2 ± 0.3	4.3 ± 0.1
3	8.6 ± 0.6	6.1 ± 2.2	3.0 ± 1.4	9.0 ± 0.0	3.7 ± 0.1	8.2 ± 0.3	8.2 ± 1.2	2.0 ± 0.3	9.1 ± 0.2	4.3 ± 0.1
4	8.4 ± 1.5	6.9 ± 0.2	2.5 ± 0.7	9.1 ± 0.2	3.7 ± 0.1	8.0 ± 0.6	8.1 ± 0.4	2.0 ± 1.4	9.0 ± 0.2	4.5 ± 0.1
5	8.4 ± 0.4	7.2 ± 0.4	2.5 ± 0.7	8.8 ± 0.1	4.0 ± 0.1	7.9 ± 1.2	7.8 ± 0.4	2.0 ± 0.7	9.1 ± 0.1	4.3 ± 0.1
6	7.9 ± 0.9	7.4 ± 0.3	2.5 ± 0.7	8.8 ± 0.4	3.7 ± 0.1	7.6 ± 0.3	7.8 ± 0.2	2.0 ± 0.7	8.9 ± 0.1	4.3 ± 0.1
7	7.7 ± 0.6	7.7 ± 0.0	2.5 ± 0.7	9.1 ± 0.3	3.7 ± 0.1	8.0 ± 0.4	8.0 ± 0.3	2.0 ± 0.7	9.3 ± 0.4	4.3 ± 0.1
8	7.6 ± 1.8	7.8 ± 0.1	2.5 ± 0.7	9.1 ± 0.2	4.0 ± 0.1	7.8 ± 0.6	7.7 ± 0.1	2.0 ± 0.7	9.2 ± 0.2	4.3 ± 0.1
9	6.8 ± 1.5	7.9 ± 0.8	2.5 ± 0.7	9.0 ± 0.1	3.7 ± 0.1	7.6 ± 1.6	7.6 ± 0.8	2.0 ± 0.7	9.1 ± 0.1	4.1 ± 0.1
10	3.3 ± 1.8	7.2 ± 0.8	2.7 ± 0.4	8.9 ± 0.3	3.7 ± 0.1	7.8 ± 1.3	7.8 ± 0.5	2.0 ± 0.7	9.2 ± 0.1	4.1 ± 0.1
11	3.5 ± 0.7	7.6 ± 0.0	2.5 ± 0.7	8.7 ± 0.0	3.7 ± 0.1	8.1 ± 0.8	7.6 ± 0.2	2.0 ± 0.4	9.3 ± 0.4	4.1 ± 0.1
12	3.3 ± 0.4	7.6 ± 0.1	2.5 ± 0.7	8.6 ± 0.0	3.7 ± 0.1	7.7 ± 0.7	7.6 ± 0.1	2.0 ± 0.7	9.2 ± 0.1	4.3 ± 0.1

Microbial counts are measured in log CFU/g. Values are the means ± standard deviations from two independent experiments

Referring to NLAB, they increased of about four and five log cycles in C1 and C2 fermentation respectively. This high value remained constant in C2 fermentation, until the end of the refreshments, while in C1 fermentation decreased after 9 days, at about 10^3^ CFU/g. The observed different trend could be related to a lower pH value measured in C1 fermentation, which decreased from 5.1 at the beginning of the fermentation to 3.7 after two refreshment steps; it could contribute to control the growth of non-acidophilic bacteria.

### Molecular characterization of microbial isolates

With the aim to characterize the natural microbial population involved in maize bran fermentation, 135 colonies of presumptive LAB were selected from different MRS plates, obtained by the analysis of the two different maize bran samples subjected to sourdough like fermentation process, as previously reported. Specifically, 68 isolates (36 cocci and 32 rods) from C1 and 67 isolates (15 cocci and 52 rods) from C2 samples were selected.

**Fig. 1:**
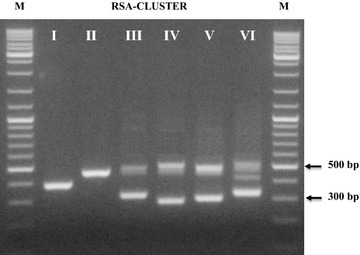
RSA profiles of representative LAB isolates of each cluster obtained. M: DNA ladder mix

**Fig. 2:**
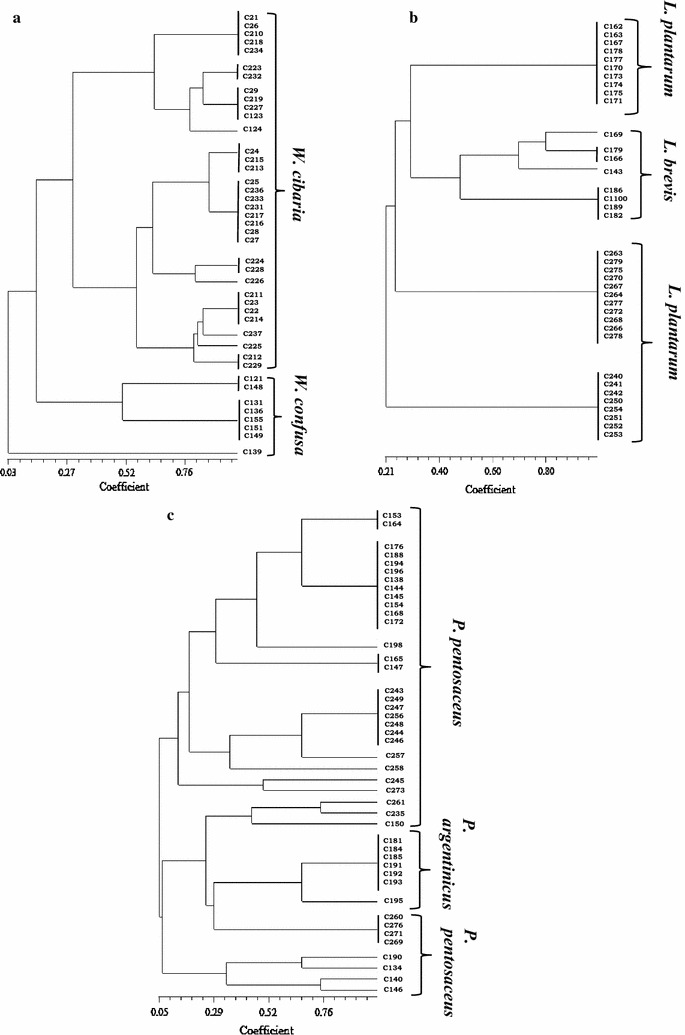
RAPD-PCR profiles of LAB isolated during maize bran sourdoughs fermentation. Primers M13 (**a**, **b**) and LP1 (**c**)

The 135 new isolates were submitted to molecular analysis for their identification. A first clustering step was reached by PCR amplification of the *16S*–*23S* rRNA spacer region (RSA). Within 51 coccal isolates, three different profiles were obtained (Fig. 1). Clusters I and II grouped three isolates each one, whose profiles were referable to *Lactococcus lactis* and *Leuconostoc lactis* species, characterized by one band migrating approximately at 380 and 460 bp, respectively; these profiles were confirmed by species-specific amplifications (Lee et al. 2000; Pu et al. 2002). Cluster III grouped the majority of the coccal isolates (88%); the RSA profile, characterized by three bands of about 310, 480, 500 bp, was associated to *Pediococcus* genus. A genus-specific PCR showed positive signals for all 45 isolates (Pfannebecker and Fröhlich 2008). Through *16S* rRNA analysis of representative strains of the cluster, specific PCR for *P. pentosaceus* (Pfannebecker and Fröhlich 2008) and a PCR assays developed in this study for identification of *P. argentinicus* (based on primer set designed within variable regions of *23S* rRNA gene (Table 1), seven strains have been ascribed to *P. argentinicus* species and the remaining 38 isolates to *P. pentosaceus*. *P. argentinicus*, isolated from Argentinean wheat flour is a described species of *Pediococcus*, showing a high phylogenetic relatedness with *P. pentosaceus* (De Bruyne et al. 2008). Figure 2a shows the RAPD-PCR banding patterns of the 45 *Pediococcus* isolates; a high degree of genetic variability can be observed.

The 84 rod isolates were grouped in three different clusters (IV, V, VI in Fig. 1). Clusters IV and V included 29 and 8 isolates respectively, whose characteristic profiles for the species *Lactobacillus plantarum* and *L. brevis* have been confirmed by species-specific amplifications (Torriani et al. 2001; Coton et al. 2008). Their RAPD profiles are presented in Fig. 2b. Differently from the high polymorphism found in *Pediococcus,* the degree of intra-species variability in *L. plantarum* was very low, despite the isolates came from two different samples of bran and from different refreshments. Cluster VI grouped 47 isolates with a RSA profile constituted by three bands of about 340, 440 and 530 bp: *16S* rRNA sequencing of a representative strain of the cluster indicated the belonging to the species *Weissella confusa.* A subsequent genus-specific PCR (Jang et al. 2002) showed positive signals for all 47 isolates. Since no species-specific probes are available for the identification of *Weissella* species, we carried out l-arabinose utilization test and a restriction analysis of the *16S* rDNA with restriction endonucleases able to underline the polymorphism existing within the gene of related species (*Alu*I, *Dra*I and *Cfo*I) (Table 3). The results obtained permitted to ascribe 12 isolates to *W. confusa* (l-arabinose negative) and 35 isolates to *W. cibaria* (l-arabinose positive), the species most phylogenetically related to *W. confusa.* Their differentiation was also obtained by RAPD-PCR experiments with primer LP1 (Fig. 2c).

**Table 3:** Restriction patterns obtained for differentiation of *Weissella* sp. (a) and yeasts (b) isolated from maize bran fermentation

*16S* rDNA (bp)	Restriction fragments (bp)		Assigned species
(Weiss_F-Weiss_R)	*Dra*I	*Alu*I	
(a)			
727	639 + 88	370 + 222 + 135	*W. cibaria*
727	727	505 + 222	*W. confusa*

ITS1-ITS4 (bp)	Restriction fragments (bp)		Assigned species
	*Hin*fI	*Taq*I	
(b)			
740	366 + 374	321 + 138 + 116 + 106	*K. unispora*
589	301 + 288	304 + 285	*W. anomalus*
725	245 + 180 + 110 + 70	300 + 245,190	*K. marxianus*

A total of 12 NLAB isolates were collected from CASO agar plates. The isolates from the refreshments of dough C1 belonged to the Gram negative *Acetobacter* genus, being *A. cibinongensis* (two strains) and *A. orientalis* (one strain) the isolated species, along with *Enterobacteriaceae*, primarily *Enterobacter asburiae* (two strains), *E. ludwigii* (one strain), *E. cloacae* (one strain) and then *Escherichia coli* (one strain). Differently, NLAB identification from the doughs of maize bran C2 demonstrated a representative presence of Gram positive bacteria belonging to *Staphylococcus* genus, being *S. warneri* (two strains) and *S. pasteuri* (one strain) the isolated species.

The next step was the identification of the 93 yeast isolates (51 from C1 and 42 from C2). Also in this case a first clustering has been obtained through the amplification of the Internal Transcribed Spacers. Four clusters were distinguishable: cluster I grouped five isolates with a PCR product of 440 bp, cluster II 32 isolates with a PCR product of 600 bp, cluster III 12 isolates with a PCR product of 720 bp and cluster IV 44 isolates with a PCR product of 740 bp. *26S* rDNA gene sequencing of representative strains of each cluster allowed to ascribe the isolates of the four clusters to the species *Pichia fermentans*, *Wickerhamomyces anomalus, Kluyveromyces marxianus* and *Kazachstania unispora,* respectively. A further digestion of the amplified ITS products with *Taq*I and *Hin*fI, permitted the obtainment of a specific restriction profile for all isolates of the same species (Table 3).

Finally, three representative fungal isolates were identified as *Mucor circinelloides*, *Mucor irregularis* and *Fusarium verticillioides*. These fungal species, causing several diseases and producing a wide range of mycotoxins, have been associated with various cereal crops and processed grains (Murillo-Williams and Munkvold 2008; Pitt and Hocking 2009).

### Dynamics of microbial population during fermentation

**Fig. 3:**
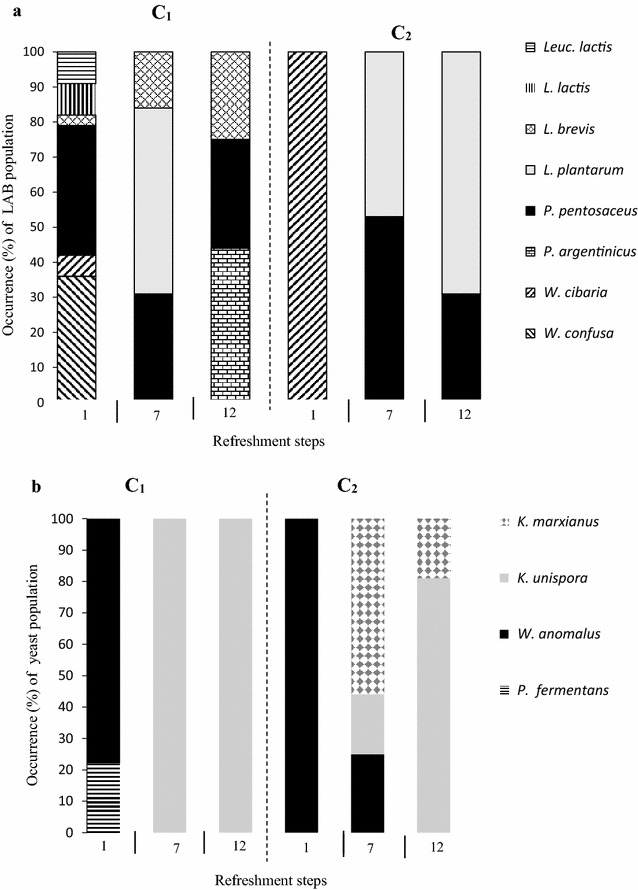
Changes in community structure of lactic acid bacteria (**a**) and yeasts (**b**) during sourdough-like fermentation of the two maize bran samples (C1 and C2)

Species composition and LAB and yeasts succession during the fermentation of the two samples of maize bran are reported in Fig. 3. As shown, the fermentation of both bran samples was characterized by a microbial succession. The initial stage of the fermentation was characterized by co-dominance of *Weissella* sp. and *Pediococcus* sp. in C1 sample and predominance of *Weissella* sp. in C2 sample. *W. anomalus* was the dominant yeast species in both fermentations. At the refreshment step 7, a natural selection was produced, with the disappearing of *Weissella* in both samples and the presence of *L. plantarum* (53% of the isolates in C1 and 45% in C2 samples), who became the main species in C2 (69%) at refreshment step 12. In C1 samples a concomitant presence of *L. brevis* was observed. *P. pentosaceus*/*argentinicus* occurred at all stage of refreshment, even if with different incidence. Regarding yeast succession, *W. anomalus* disappeared from the community, while *K. unispora* was subsequently detected. In C1 fermentation this was the only yeast found at the end of the fermentation; in C2 sample a significant presence of *K. marxianus* (56% and 19% at refreshment steps 7 and 12 respectively) was also observed.

### Characterization of native maize bran and fermented bran

**Fig. 4:**
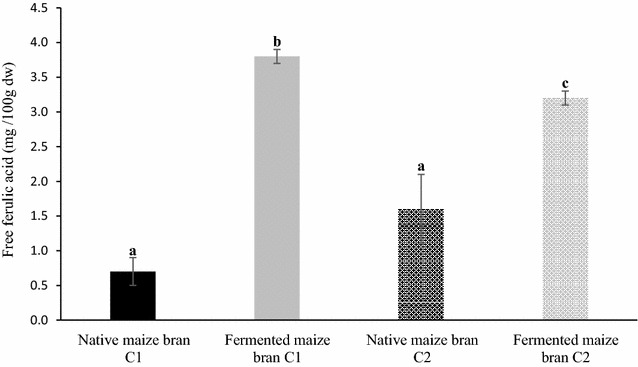
Free ferulic acid (mean ± SD; expressed as mg/100 g of dry weight) content in native and fermented (refreshment 12) brans. Bars not sharing common letters are significantly different (*p* < 0.05)

**Fig. 5:**
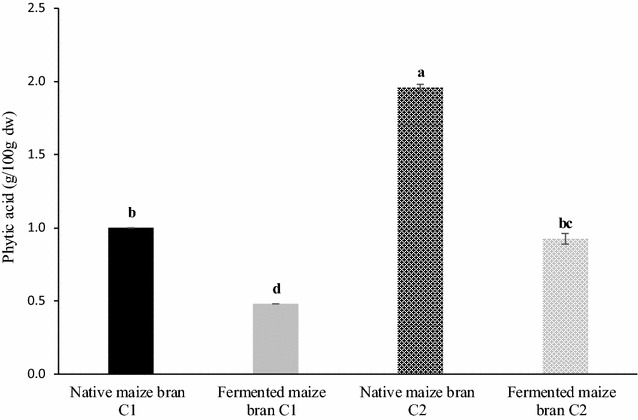
Phytic acid (mean ± SD; expressed as g/100 g of dry weight) content in native and fermented (refreshment 12) brans. Bars not sharing common letters are significantly different (*p* < 0.05)

Table 4 reports the proximate composition detected in native and fermented samples. In native maize bran C2, ash, protein and sugars content was significantly (*p* < 0.05) higher than in C1, differences that could probably be related to cultivars and pedo-climatic conditions (Sokrab et al. 2011). At the end of the sourdough-like fermentation process (refreshment 12), both the tested brans contained slightly higher amounts of lipid and significantly lower (*p* < 0.05) protein levels from native sample only for C2. As expected, sourdough-like fermentation resulted in a significant (*p* < 0.05) decrease of the total content of sugars. On the other hand, this process induces a threefold significant (*p* < 0.05) increase in soluble dietary fiber in both C1 and C2 samples, without affecting their insoluble fraction. Regarding TOTAX, the results obtained suggest that bran sourdough-like fermentation contribute to their solubilization, with a significant (*p* < 0.05) reduction of this hemicellulosic fraction, accompanied only by a slight increase of WEAX. The arabinose to xylose ratio in WEAX, an indicator of the average degree of arabinose substitution (avDAS), showed a little reduction, indicating that, in both fermented brans, WEAX were slightly less substituted with arabinose than those present in the native brans (Damen et al. 2011; Broekaert et al. 2011). The levels of FFA detected in C1 and C2 maize bran before and after sourdough-like fermentation are shown in Fig. 4. The concentration of FFA assessed in native brans was not statistically different (0.7 ± 0.2 and 1.6 ± 0.4 mg/100 g for C1 and C2 respectively) and showed a significant (*p* < 0.05) increase after the sourdough-like fermentation (two- and fivefold in C2 and C1 respectively). Phytate is known to chelate several nutritionally essential nutrients and can negatively influence the activity of digestive enzymes by the chelation of mineral cofactors or by interacting with protein. In accordance with our previous study on wheat (Manini et al. 2014) the sourdough-like fermentation process promoted a degradation of phytic acid (Fig. 5) also in maize brans. In particular, this antinutrient compound was significantly reduced of about 50% in both C1 and C2, likely through the activation of microbial and endogenous phytases.

**Table 4:** Chemical composition (mean ± SD; expressed as % of dry weight) of native and fermented (refreshment 12) brans

	Native maize bran C1	Fermented maize bran C1	Native maize bran C2	Fermented maize bran C2
Ash	0.9 ± 0.0a	1.1 ± 0.0a	2.2 ± 0.1b	1.8 ± 0.2c
Proteins	6.4 ± 0.3a	7.2 ± 0.2a	9.0 ± 0.3b	7.7 ± 0.5a
Lipids	2.7 ± 0.0	4.2 ± 0.1	3.5 ± 0.8	4.6 ± 0.2
Sugars	1.7 ± 0.1a	0.2 ± 0.0b	4.3 ± 0.4c	0.5 ± 0.0b
Glucose	0.3 ± 0.0	0.2 ± 0.0	2.6 ± 0.2	0.2 ± 0.0
Fructose	0.3 ± 0.0	nd	1.4 ± 0.2	0.2 ± 0.0
Sucrose	1.2 ± 0.1	nd	nd	nd
Maltose	nd	nd	0.4 ± 0.0	nd
Insoluble fiber	35.2 ± 3.0	36.0 ± 1.8	41.2 ± 3.7	45.6 ± 1.6
Soluble fiber	0.6 ± 0.1a	1.7 ± 0.4b	0.7 ± 0.2a	2.3 ± 0.3b
Total arabinoxylans (TOTAX)	20.1 ± 1.5a	13.1 ± 0.1b	21.3 ± 1.1a	10.6 ± 1.2b
Arabinose/xylose in TOTAX	0.75	0.74	0.70	1.28
Sol. arabinoxylans (WEAX)	0.1 ± 0.0	0.3 ± 0.0	0.1 ± 0.0	0.2 ± 0.0
Arabinose/xylose in WEAX	1.43	1.07	2.98	2.35

Data in the same row not sharing common letters are significantly different (*p* < 0.05)

*nd* not detectable

## Discussion

The continuous search for functional ingredients providing health effects and the possibility to take advantage of agro-industrial by-products have attracted great interest in using bran-enriched products. In this research, a polyphasic approach has been carried out to characterize at microbiological and chemical level maize bran in order to enhance its technological and nutritional properties.

Little data are available in literature on microbial composition of maize and maize bran: to our knowledge up to now researches were carried out on the evolution of fermenting microbiota in wheat or rye brans (Katina et al. 2007; Manini et al. 2014) and in ethnic food products, such as tarhana (Settanni et al. 2011). Very lacking information exists on microbial population during maize bran fermentation. In our study, microbial population colonizing the first steps of natural fermentation of maize bran is characterized by a predominance of species belonging to the *Pediococcus* and *Weissella* genus. These physiological groups have also been selected during maize-based spontaneously fermented doughs produced in West African countries (Oguntoyinbo et al. 2011; Okeke et al. 2015; Assohoun-Djeni et al. 2016). Even if little data are available, this microbial population could be considered maize/maize bran endogenous.

During daily propagation of sourdoughs, *L. plantarum* became the main LAB species. *L. plantarum* is a versatile and competitive species, that can adapt to different environmental conditions (Minervini et al. 2010) and these features can explain its presence, as member of the complex microbiota, in many sourdoughs (De Vuyst et al. 2014). The interest dedicated to this species, particularly in the bakery sector, is related to the ability of producing antimicrobial compounds, especially antifungal metabolites (Crowley et al. 2013).

During the maize bran fermentation, we found several species of yeasts, some of who predominate in the first step of fermentation, as *W. anomalus*, other at the end of the refreshments, as *K. unispora*. This is the first report on association between LAB and yeasts in maize bran fermentation process. *W. anomalus* was shown to inhibit the growth of fungi in airtight-stored cereal grains (Coda et al. 2011). Its presence in maize bran sourdoughs, in association with *L. plantarum* could explain the decrease of vital molds we observed during fermentation. *K. unispora* plays a significant role both in cheese ripening and in fermented milk production; it has been documented to exist in few sourdough ecosystems, as artisan wheat and rye sourdough albeit in low abundance (Bessmeltseva et al. 2014). On the contrary, our data suggest that this species can be considered representative of the yeast microbiota selected during maize bran refreshment steps.

Evident differences in dominant NLAB were found between C1 and C2 samples; this could be a consequence of differences in the environmental conditions (i.e., temperature, water, and maize provenance) and in the evolution of dough pH; this parameter has a strong and decisive influence on the control and selection of bacteria. Among the identified NLAB, the family *Enterobacteriaceae* was the predominant in C1 doughs, followed by *Acetobacter* genus, differently from the doughs C2 where *Staphylococcus* genus was the prevalent. Regarding *Enterobacteriaceae,* strains of *E. asburiae* were previously identified among thermotolerant wheat associated bacteria from a peninsular zone of India (Verma et al. 2016) and one *E. ludwigii* strain isolated from *Lolium perenne* rhizosphere showed plant growth promoting properties (Shoebitz et al. 2009). The genus *Acetobacter* has been previously reported to dominate, together with the genus *Lactobacillus,* the first step of fermentation of whole crop maize silage (Sträuber et al. 2012). Different species of the genus *Acetobacter* were also found in a preliminary study to explore the bacterial microbiota in Colombian maize fermented dough *Masa Agria* (Chavez-Lopez et al. 2016). Lactic and acetic acid provide sour taste in maize, but, at the same time, these fermentation products promote the activation of the phytases naturally present in grains and in bacteria, by a decrease of the pH value. The action of phytases plays a key role in improving nutritional value by increasing the bioavailability of essential dietary minerals. In addition, as it was observed in this study, *Acetobacter* species can be present in the later stage of cereal fermentation for their ability to utilize molecules other than sugars and to tolerate low pH conditions (Chavez-Lopez et al. 2016). Our findings on *Staphylococcus* isolated from C2 doughs are in agreement with other researchers who stated that *Staphylococcus* sp. bacteria were isolated and identified from fresh yellow grains and roots of maize in Nigeria (Orole and Adejumo 2011). Their persistence during the fermentation steps can be explained by the higher pH values profile of C2 doughs (4.3–4.1) that did not lead to selective environmental conditions.

The pH reduction during the process activated the enzymatic activities related to the microbial consortium characterizing spontaneous maize bran fermentation, providing a “destructuration” of the fiber fraction and a reduction of phytic acid content in both the tested brans. Moreover, the fermentation process provided for an increase in the soluble dietary fiber that can be attributed to microbial exo-polysaccharides production, as evidenced in previous studies on wheat and sorghum sourdoughs (Galle et al. 2010; Ganzle 2014). Our results also suggest that bran fermentation promoted AX solubilization since the percentage of WEAX/TOTAX was increased from 0.5% (native bran) to 2% (fermented bran). The data obtained appears interesting from a nutritional point of view, since soluble fiber positively affect post-prandial glucose metabolism and satiety (Anderson et al. 2009; Dikeman and Fahey 2006).

As already reported (Katina et al. 2006), endogenous and microbial xylanases could have a key role in fiber solubilization. Xylanase activity has been found in a *P. fermentans* strain (Madrigal et al. 2013), in *Weissella* spp. strains isolated from Indian fermented foods (Patel et al. 2013) and in *L. plantarum* and *P. pentosaceus* starter cultures (Laitila et al. 2006). These microorganisms were detected in all refreshment steps, suggesting a potential role of their enzymatic pool in increasing WEAX concentration, as assessed in fermented brans. Moreover, even if maize bran has been reported as a substrate resistant to enzymatic digestibility (Agger et al. 2010), our results suggest an AX “destructuration”, during maize bran fermentation. These data, interesting from a functional/nutritional point of view, deserve to be deepened by further studies.

Ferulic acid is a bioactive antioxidant compound abundant in maize bran (Zhao and Moghadasian 2008), where it is strictly cross-linked to arabinose residues in AX (Saulnier and Quemener 2009). The suggested destructuration of cell walls material, could also partly explain the increased FFA content that we assessed in fermented brans. In addition to microbial endoxylanases, other degrading enzymes, such as arabinofuranosidases, feruloyl esterases, acetyl esterases, and alpha-glucuronidases, can be involved (Grootaert et al. 2007). Even if these enzymatic activities are poorly studied in *Lactobacillus* genus, a few papers show the presence of feruloyl esterases in *L. plantarum* strains (Esteban-Torres et al. 2015) and of arabinofuranosidases in selected strains of *L. brevis* (Michlmayr et al. 2013).

Phytic acid is the major storage form of phosphorous comprising 1–5% by weight in cereals, located in bran fraction and pericarp in rice and wheat, and found mainly in the germ of maize kernels (Schlemmer et al. 2009). From a nutritional point of view, this compound is considered an antinutritive factor, for its ability to chelate dietary minerals, thus reducing their bioaccessibility and bioavailability. Our data show that the fermentation process promote a significant reduction of phytic acid, likely related to an activation of endogenous phytases and to the presence of specific microbial biotypes able to produce significant extracellular phytase activities.

In conclusion, the sourdough-like fermentation of maize bran seems a suitable bio-approach in promoting nutritional and functional properties of this cereal by-product. Further studies are ongoing for the characterization of the isolated microorganisms and for understanding their role in specific activities related to the enhancement of the properties of fermented maize bran as well as their safety. These studies will allow the selection of starter cultures, according to their metabolic and enzymatic activities, in order to conduct “tailored” fermentation process and improve brans or whole-meal flours from nutritional, functional and technological points of view.

## Abbreviations

LAB: lactic acid bacteria
A.I.R.E.S: Associazione Italiana Essiccatori Raccoglitori Stoccatori di Cereali e Semi oleosi
AX: arabinoxylans
EFSA: European Food Safety Authority
WEAX: water-extractable arabinoxylans
AXOS: arabino-xylanoligosaccharides
XOS: xylan-oligosaccharides
TOTAX: total arabinoxylans
NLAB: non-lactic acid bacteria
PCR: polymerase chain reaction
RSA: *16S*–*23S* rDNA spacer region
rDNA: ribosomal DNA
ITS: Internal Transcribed Spacer
BLAST: Basic Local Alignment Search Tool
RAPD: Random Amplification of Polymorphic DNA
ANOVA: analysis of variance
CFU: colony forming units
MRS: de Man, Rogosa, Sharpe
CASO: Casein-peptone Soymeal-peptone
avDAS: average degree of arabinose substitution
FFA: free ferulic acid
